# Genetic diversity of bacterial endophytes associated with *Crotalaria* (Rattlepods)

**DOI:** 10.1371/journal.pone.0362184

**Published:** 2026-11-11

**Authors:** Edinah Ongaga, Johnstone O. Neondo, Joshua Kiilu Muli, Peter K. Kamau, Christine Bii, Eddy Odari, Nancy L. M. Budambula

**Affiliations:** 1 University of Embu (UoEm), Embu, Kenya; 2 Jomo Kenyatta University of Agriculture and Technology (JKUAT), Nairobi, Kenya; 3 South Eastern Kenya University (SEKU), Kitui, Kenya; 4 Kenya Medical Research Institute (KEMRI), Nairobi, Kenya; University of Duhok, IRAQ

## Abstract

*Crotalaria* is a diverse legume genus valued for its ecological and agronomic roles. However, its associated endophytic microbiome remains minimally characterized. This study examined the genetic diversity and community structure of bacterial endophytes inhabiting domesticated and wild *Crotalaria* species. Twenty-nine healthy *Crotalaria* plant samples were analyzed using the culture-independent 16S rRNA gene amplicon sequencing approach. Genomic DNA was extracted using the Phenol-Chloroform method from both domesticated and wild samples. Sequencing of 16S ribosomal RNA gene was performed using Illumina MiSeq to profile endophytic bacterial communities. The sequence data was imported into R and processed using Divisive Amplicon Denoising Algorithm 2 (DADA2) pipeline. In total, 1,546,153 high-quality sequence reads were obtained and grouped into 611 amplicon sequence variants (ASVs) at 97% sequence similarity threshold. Domesticated samples exhibited higher observed species richness (median of 39 ASVs) than wild samples (median of 36 ASVs), while wild samples showed greater microbial community evenness (median ~1.08) compared to domesticated samples (median ~0.93). Beta diversity analyses showed clear separation between domesticated and wild endophytic bacterial communities, with domestication status explaining most of the observed variations (PERMANOVA: Bray-Curtis R^2^ = 0.826; Jaccard R^2^ = 0.846), but PERMANOVA detected no statistically significant difference (Bray-Curtis and Jaccard; *p* = 0.067). Overall, five bacteria phyla were detected in the *Crotalaria* samples. Pseudomonadota was the dominant phylum (64.27%) followed by Cyanobacteriota (35.5%). *Enterobacteriaceae* was the most abundant family (81.06%) with *Enterobacter* emerging as the most abundant genus in both domesticated (38.3%) and wild (89.4%) accessions. Notably, *Pseudomonas* was relatively enriched in domesticated plants while several bacterial genera were restricted to either wild or cultivated hosts, indicating habitat-associated endophytic specialization. This study provides the first comprehensive assessment of endophytic bacterial diversity in Kenyan *Crotalaria* species and highlights wild germplasm as an important reservoir of microbial diversity with potential biotechnological value.

## 1. Introduction

Plants are often colonized by a wide range of microorganisms called endophytes, which reside in their internal tissues and organs without causing any visible harm [1,2]. Plant-associated microbiomes are now recognized as integral components of host biology, shaping nutrient acquisition, stress tolerance and disease resistance. The most common endophytes in plants are bacteria and fungi [3]. Numerous factors including plant species, plant organ, microbial strain and ecological conditions influence the success of endophytic colonization [4]. Generally, endophytes are more abundant in plant roots compared to stems or leaves [2,5]. To ensure stable association, endophytes promote plant growth and tolerance to environmental stress [1]. Within the host plants, endophytes also take part in metabolic and physiological activities, such as; nitrogen fixation, nutrient uptake and plant pathogen control [2]. Endophytic microbiota is a promising source of many bioactive compounds that are useful in medicine, biotechnology and agriculture [1]. In order to fully exploit the ecological value and biotechnological potential of endophytic microorganisms inhabiting plant tissues, it is crucial to understand their diversity and spectrum. Emerging evidence suggests that plant domestication is a major driver of microbiome assembly, often reshaping microbial composition and function without necessarily reducing overall diversity [6,7]. Cultivation practices including fertilization, irrigation and host selection can alter microbial recruitment, leading to shifts in dominant taxa and community structure. Wild plant populations have been shaped by long-term co-evolution in heterogeneous environments thus may harbour more stable and functionally balanced microbial consortia [8].

*Crotalaria* is one of the largest angiosperm genera with approximately 700 species of which 540 species are found in Africa [9]. Although members of this genus have been reported in nearly all agroecological zones in Africa, majority occur in tropical eastern and southern regions [9–11]. *Crotalaria* is considered a multi-purpose legume/crop in agricultural production systems. It is mostly grown for food, weed control and restoration of soil fertility. *Crotalaria brevidens* Benth, *C. ochroleuca* G. Don and *C. trichotoma* Bojer are the three species that are eaten as vegetables in Africa*.* They are rich in calcium, iron, phosphorous, proteins, vitamin A and Vitamin C [10,12]. Other edible species of *Crotalaria*, include *C. lachnophora* A.Rich, *C. quinquefolia* L., *C. retusa* L, *C. longirostrata* Hook. & Arn., *C. pallida* Aiton, *C. cajanifolia* Kunth., *C. macrocalyx* Benth., *C. natalitia* Meisn., *C. spectabilis* Roth, *C. juncea* L, *C. tetragona* Roxb. Ex Andrews and *C. micans* Link [10].. *Crotalaria* species have also been utilized in indigenous medicinal uses. Members of this genus contain diverse phytochemicals including alkaloids, flavonoids, triterpenoids, phenols, quercetins and/or β-sitosterol, some of which have been shown to have therapeutic potential [12–15]. In addition to their medicinal and agronomic benefits, *Crotalaria* species harbor diverse microorganisms in their inner tissues, including endophytic bacteria which are potential sources of bioactive secondary metabolites for use in medicine and agriculture.

The assessment of the taxonomic diversity of endophytic bacteria is crucial in unraveling the complex plant-endophyte evolutionary interactions that have shaped the host fitness and ecological adaptation [16,17]. Comprehending these complex associations can prompt the creation of innovative strategies for conservation agriculture, such as the use of ancestral microbial assemblages to promote health and productivity in plants [17]. Globally, plant endophytes have been the subject of extensive research, but little is known about their composition, activities and function [18,19]. Conventional culture-based methods have been instrumental in isolating microbial strains, but they only capture less than 10% of the total microbial diversity due to the unculturable nature of most bacteria [20]. Although this traditional approach is crucial in isolating single microbial strains that can be used in conservation agriculture, next-generation sequencing (NGS) methods are widely recognized and are considered highly efficient for structural and functional profiling of endophytic microbial communities in different plants since they target all the bacteria, including the unculturable species [21].

Next-generation sequencing (NGS) technologies, such as Illumina, Ion Torrent, Pacific Biosciences (PacBio), ABI SOLiD, Ion Personal Genome Machine (PGM) and Oxford Nanopore, have significantly advanced wet laboratory protocols, test kits and bioinformatic pipelines in plant-microbiome research [19,22,23]. Such advancements have reduced the cost and time associated with understanding the structure and function of endophytic microorganisms found in plants, while maintaining high accuracy [19]. The basis of NGS platforms for microbial diversity studies is 16S rRNA gene, a universal prokaryotic marker. The 16S rRNA gene has several highly conserved and hyper-variable regions important for taxonomic classification and separation [24]. Among the various NGS platforms currently in use, Illumina MiSeq machine, which supports high-throughput paired end sequencing, has been widely employed in microbial community studies [21,22]. In microbial diversity studies, the Illumina platforms primarily target the V3-V4 hypervariable areas in the 16S rRNA gene [21]. Advances in high-throughput sequencing, particularly 16S rRNA gene amplicon profiling coupled with denoising algorithms such as DADA2, have transformed our ability to resolve microbial communities at fine taxonomic scales, moving beyond traditional operational taxonomic units toward more precise amplicon sequence variants (ASVs) [25,26].

Previous studies have reported that *Crotalaria* species are inhabited by diverse bacterial endophytes with biotechnological and ecological benefits. In *Crotalaria pumila*, *Methylobacterium* sp. Cp3 was identified as the predominant bacterial endophyte across three seed generations [27]. Aserse *et al*. [28] recovered endophytic bacterial strains belonging to seven genera including *Agrobacterium, Burkholderia, Paenibacillus, Pantoea, Pseudomonas, Rhizobium* and *Serratia* from root nodules of *Crotalaria incana*. Majority of the isolates showed plant growth promoting properties *in-vitro*. Similarly, an endophytic bacteria *Bacillus velezensis* strain G2T39 isolated from *C. retusa* demonstrated biocontrol activity against *Fusarium* and *Colletotrichum* species [29]. However, majority of the endophytic diversity studies in *Crotalaria* have focused on isolation and characterization of individual strains or specific *Crotalaria* plant species or tissue. As a result, community-level diversity and composition of endophytic bacteria associated with domesticated and wild *Crotalaria* species in Kenya remains poorly characterized. Despite growing recognition of the role of plant domestication in shaping microbial communities associated with plants, it is still not clear whether domestication alters richness, composition and diversity of endophytic bacterial communities inhabiting Kenyan *Crotalaria* germplasm. We hypothesized that cultivated *Crotalaria* morphotypes would differ in endophyte composition from wild-type morphotypes grown under the same conditions. Additionally, it was expected that known nitrogen-fixing and plant-growth-promoting bacterial genera, would form the core of the community, as reported in other legumes [30]. The research questions addressed by the study were: (i) What is the taxonomic composition and community diversity of bacterial endophytes living in association with domesticated and wild *Crotalaria* plants? (ii) Does endophytic bacterial alpha and beta diversity differ between domesticated and wild *Crotalaria* plants? and (iii) Does domestication status influence the composition and relative abundance of the bacterial endophytes living in association domesticated and wild *Crotalaria* plants?

## 2. Materials and methods

### 2.1. Study site and *Crotalaria* plant sample collection

All the plants used in this study came from the *Crotalaria* germplasm collection described by Muli *et al*. [31]. The collection comprised of 80 accessions of 21 species collected from five regions of Kenya and the Genetic Resources Research Institute of Kenya (GBK). In that earlier study, the cultivated species, *C. brevidens*, *C. ochroleuca* and *C. trichotoma* were treated as domesticated, and therefore the term “domesticated” in the current study is used in a similar manner, to refer to *Crotalaria* species grown as cultivated vegetables. All the other species which are not grown as crops were referred to as “Wild”. Seeds were sown in experimental plots at the University of Embu Farm, Embu County, Kenya (approximately 0°32′ S, 37°27′ E; 1494 m above sea level). The soils are predominantly humic Nitisols derived from volcanic rocks. The area has a bimodal rainfall pattern, with long rains received between March and June, and short rains between October and December. All morphotypes were grown at this one site, under rain-fed conditions and no fertilizer application.

Plants were grouped into six morphotypes (E1–E6) by leaf and pod characters. E1 (slender pod, n = 8), E2 (broad pod, n = 11), E3 (trifoliate-hairy, n = 4), E4 (trifoliate non-hairy herb, n = 2), E5 (trifoliate non-hairy shrub, n = 2), and E6 (multifoliate, n = 2). A total of 29 healthy plants, representing 21 species and eight varieties of some of these species, were sampled three months after sowing. Plants showing no wilting, discolouration or leaf spots were chosen purposively from the plots within each morphotype. From each plant, leaf, stem-bark and root tissue was cut with a sterile blade and bagged separately. Root nodules were excluded, prior to DNA isolation from the roots. After surface sterilization, the three tissues from each plant were combined into one composite sample, and DNA isolated from plants of the same morphotype were pooled before sequencing.

### 2.2. Surface sterilization

Leaf, stem-bark and root tissues of each of the 29 plant samples were surface sterilized separately. The collected plant tissues were initially cleaned with running tap water to remove any surface residue. They were then surface sterilized as described by War Nongkhlaw & Joshi [32], with minor modifications. The plant tissues were immersed sequentially in sterile distilled water for 1 minute, 70% ethanol for 30 seconds, 3.5% sodium hypochlorite solution for 2 minutes, 70% ethanol for 30 seconds and final rinsing with sterile water three times. All sterilized tissues were dried in pre-sterilized filter paper in a laminar flow hood. For each individual plant, the leaf, stem-bark and root tissues were combined and subsequently processed as a composite sample for DNA extraction. To confirm the success of the sterilization procedure, surface-sterilized plant tissues were imprinted onto nutrient agar (NA, Oxoid), incubated for 10 days and observed for growth of contaminants.

### 2.3. Total DNA isolation

Plant DNA was isolated from the *Crotalaria* plant samples as described by Sambrook *et al*. [33] with minor adjustments. Approximately, 0.5 g of the composite sample comprising of root, stem and leaf material from each of the 29 *Crotalaria* plants was frozen in liquid nitrogen and pulverized to a fine powder using a sterilized, precooled pestle and mortar. Thereafter, the fine powder was suspended in 1 ml of 1X PBS, Phosphate Buffered Saline, (containing 800 ml sterile distilled water, 200 mg KCL, 144 mg Na_2_HPO_4_, 245 mg KH_2_PO_4_, pH 7.4 supplemented with 133 mg of CaCl_2_.2H_2_0 and 200 mg of MgCl_2_.6H_2_O) and mixed by inverting the tube several times. The tube was then incubated for 3 hours at 28°C in a water bath. One milliliter of the homogenate was transferred into 2 ml microfuge tubes, centrifuged for 10 minutes at 16,400 × g and the supernatant discarded. The pellet was re-suspended in 200 μl solution A (100 mM EDTA, pH 8.0 and 100mM Tris-HCl, pH 8.0) and 10 μl of the 20 mg/ml lysozyme added. The mixture was then incubated for 30 minutes at 37°C in a water bath. Thereafter, 400 μl solution B/ lysis buffer (100 mM EDTA, pH 8.0; 100 mM Tris-HCl, pH 8.0 and 1% sodium dodecyl sulfate) was added and the tubes incubated for 10 minutes at room temperature. After that, 15ul 6M guanidinium isothiocyanate (GITC) was added, gently mixed and then incubated for 1 hour at 65°C in a water bath. After adding an equivalent volume of chloroform/isoamyl alcohol (24:1), the mixture was centrifuged for 5 minutes at 4°C at 16,400 × g. The supernatant was poured into new microfuge tubes. Thereafter, 150 μl of 7.5M ammonium acetate and an equivalent volume of isopropyl alcohol (supernatant + ammonium acetate) was added and the tubes were gently inverted to mix the samples. The tubes were centrifuged for 10 minutes at 16,400 × g and the supernatant carefully discarded. After washing the resulting DNA pellets in 300 μl ice cold 70% molecular ethanol, they were centrifuged for 1 minute at 16,400 × g and the supernatant was discarded. The tubes were inverted on paper towels on a laboratory workstation to allow DNA pellets to air dry at room temperature. The DNA was re-suspended in 30 μl of nuclease-free water, screened using 0.8% agarose gel electrophoresis, and subsequently stored at −20°C.

### 2.4. 16S rRNA gene sequencing

The DNA extracted from the 29 *Crotalaria* plants were pooled into six molecular samples (E1-E6). E1 and E2 were domesticated *Crotalaria* morphotypes, while E3, E4, E5 and E6 were wild *Crotalaria* morphotypes.

As previously described by Caporaso *et al*. [34] PCR primers 515F (GTGCCAGCMGCC GCGGTAA) and 806R (GGACTACHVGGGTWTCTAAT) were used to amplify the 16S rRNA gene V4 hypervariable region using a HotStarTaq Plus Master Mix Kit (Qiagen, USA). Amplification conditions were; pre-denaturation at 94°C for 3 minutes, denaturation at 94°C for 30 seconds, annealing at 53°C for 40 seconds, elongation at 72°C for 1 minute, 30 cycles and final elongation at 72°C for 5 minutes. Agarose gel (2%) was used to assess the quality of the amplification products. A DNA library was generated from the purified and pooled amplification products according to Illumina TruSeq DNA library preparation protocol [35]. Sequencing was done at MR DNA (Shallowater, TX, USA; www.mrdalab.com) on an Illumina MiSeq platform as guided by the manufacturer’s instructions.

### 2.5. Analysis of sequence data

The raw FASTQ files received from the sequencing facility (www.mrdnalab.com, MR DNA, Shallowater, TX) were first trimmed of primers using Cutadapt v5.2 [36]. The clean sequence reads were imported into R v4.2.0 and processed using Divisive Amplicon Denoising Algorithm 2 (DADA2) pipeline [37]. The sequences were filtered and trimmed to eliminate short, low-quality reads and chimeras. The resulting dataset including; ASV table, taxonomy table, sample metadata and phylogenetic tree were combined, normalized, and converted into a phyloseq object for analysis. Contaminant ASVs identified by decontam were eliminated and the resulting data was rarefied to the smallest sequencing depth [38]. Low-abundance and low-prevalence taxa were removed using minimum read cutoffs (≥50 total reads) and prevalence thresholds (present in ≥5% of samples). The remaining counts were transformed to relative abundances for compositional and diversity analyses performed with phyloseq and microbiome packages in R v4.2.0 [39].

### 2.6. Microbial taxonomic classification, statistical and diversity analysis

Alpha diversity was assessed using observed ASVs and Shannon index. On the other hand, Beta diversity was evaluated using Jaccard and Bray-Curtis distance metrics and illustrated with Principal Coordinates Analysis (PCoA). The PERMANOVA test ascertained the significant differences between the domesticated and wild *Crotalaria* groups. It analyzed the multivariate data by comparing the endophytic bacterial communities across all samples and subsequently produced group significance plots based on 999 permutations. Bacterial taxonomy was assigned at a 97% similarity threshold using a Naïve Bayes classifier trained by SILVA 138-99 reference database. Amplicon Sequence Variants classified as chloroplast or mitochondria, and those not assigned to Bacteria, were removed. The sequences were compared with reference sequences and interactive plots prepared to display the relative abundances of microorganisms at various taxonomic levels. The interactive plots provided the foundation for determining and comparing endophytic microbial community structure.

Since DNA was pooled by morphotype, with one group containing two (domesticated) and the other four (wild-type) pooled samples, this limited biological replication and the ability to test differences statistically. Because the small sample size and limited number of possible permutations restricted statistical power, group differences are reported descriptively rather than by statistical significance. Bray–Curtis and Jaccard dissimilarities were used for exploratory PCoA plots to visualise differences in sample composition.

### 2.7. Research approval

The research was approved by the National Commission for Science, Technology & Innovation (NACOSTI), Kenya and the research license No: NACOSTI/P/20/3594 was issued.

## 3. Results

### 3.1. Taxonomic assignment of sequences

High-throughput Illumina sequencing yielded 1,681,567 raw reads, of which 1,546,153 high-quality sequence reads were recovered after quality control and chimera removal across six *Crotalaria* samples. Denoising resolved 611 amplicon sequence variants (ASVs) at a 97% similarity level, which were taxonomically assigned using the SILVA database, spanning 5 phyla 20 families and 30 genera. The rarefaction curves (Fig 1) for all six samples plateaued at sequencing depths of 15,000–20,000 reads, indicating sufficient sampling coverage of the endophytic bacterial communities in *Crotalaria* plant samples.

**Fig 1:**
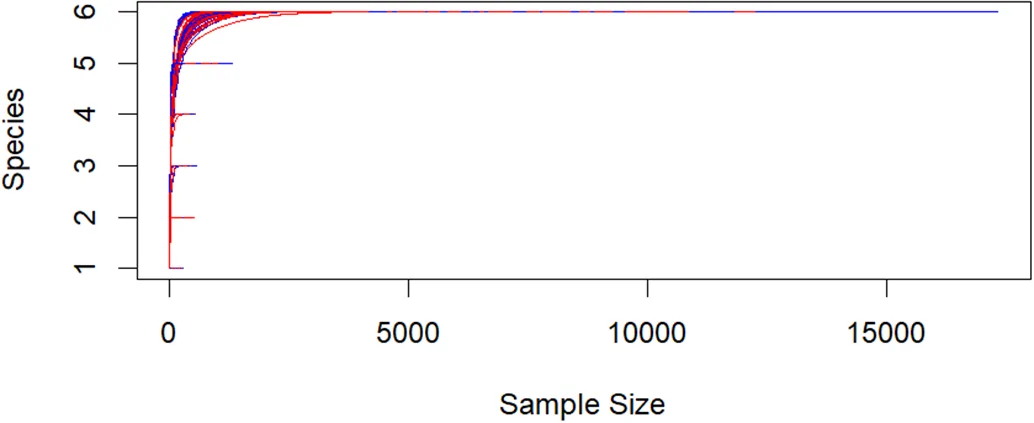
Amplicon sequence variant (ASV) rarefaction curves at varying sequencing depths in domesticated and wild *Crotalaria* samples.

### 3.2. Alpha diversity analysis

Domesticated *Crotalaria* samples recorded a higher observed species richness, with a median of 39 ASVs (IQR: 37–42), compared to wild *Crotalaria* samples, with a median of 36 ASVs (IQR: 32–40) (Fig 2a and b). In contrast Shannon diversity index was slightly higher in wild samples (median ~1.08) compared to domesticated samples (median ~0.93) indicating greater community evenness.

**Fig 2:**
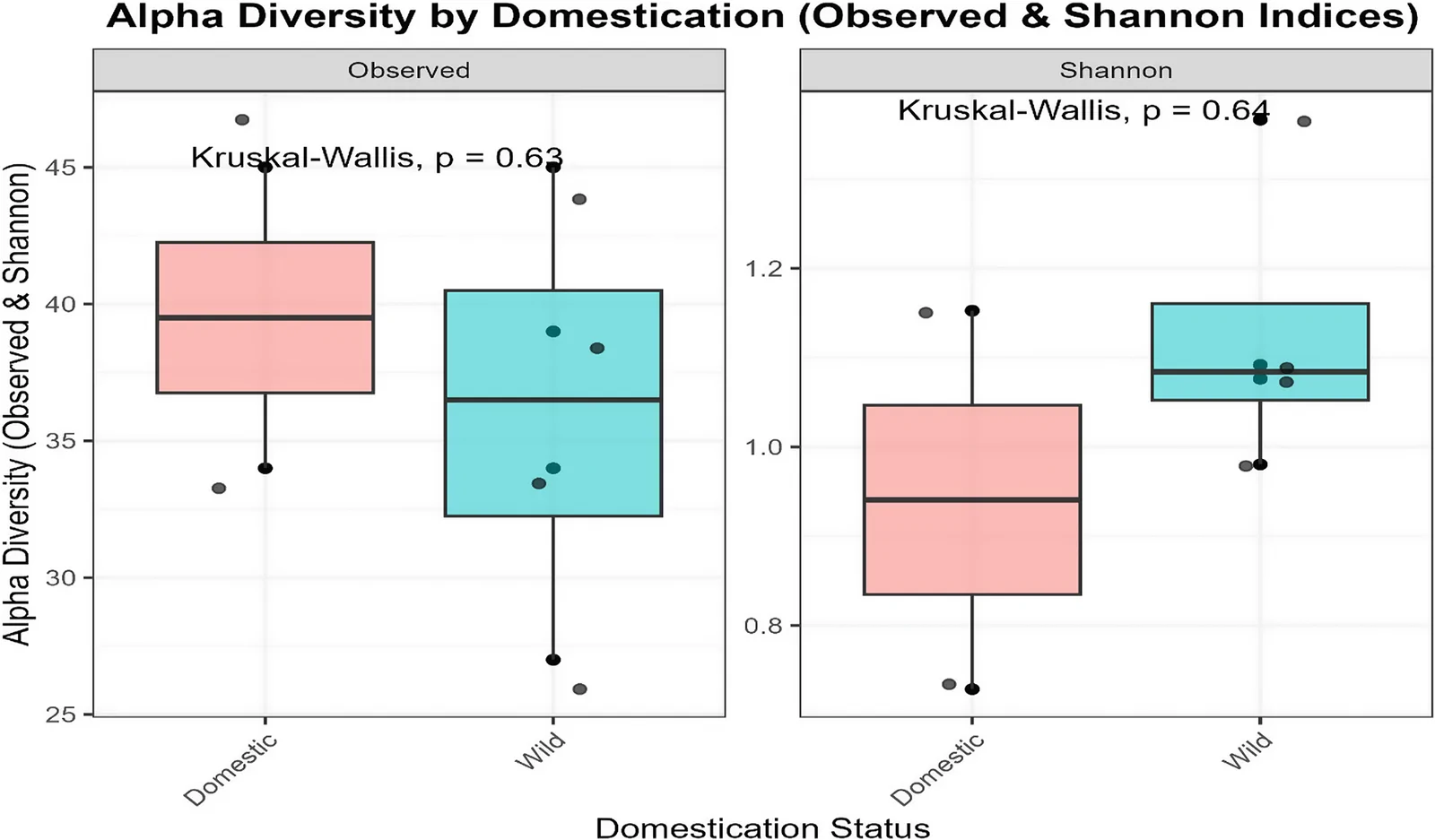
Boxplot showing alpha diversity metrics for domesticated and wild *Crotalaria* plants.

The statistical significance of the alpha diversity of microbial communities in both wild and domesticated samples was assessed using the Kruskal-Wallis test. According to the test results, no statistically significant differences were observed between domesticated and wild *Crotalaria* samples for either observed richness or Shannon diversity (*p =* 0.13). Therefore, although domesticated samples recorder higher observed richness and wild samples recorded a slightly higher Shannon diversity, these observed differences were not supported statistically.

### 3.3. Beta diversity analysis

The composition of the microbial communities in the six pooled samples (E1- E6) was compared using Bray-Curtis and Jaccard dissimilarities and displayed graphically by principal coordinates analysis (PCoA; Fig 3). The Bray-Curtis ordination (Fig 3A) accounted for 99.3% of the variation, with the first two axes explaining 88.9% and 10.4% of the variation, respectively. The domesticated group (E1 and E2) were placed at about −0.5 on axis 1. The wild samples E4, E5 and E6 were placed between 0.3 and 0.4, while E3 was placed in between. E1 and E2 were split on axis 2, while E4, E5 and E6 clustered together in the ordination.

**Fig 3:**
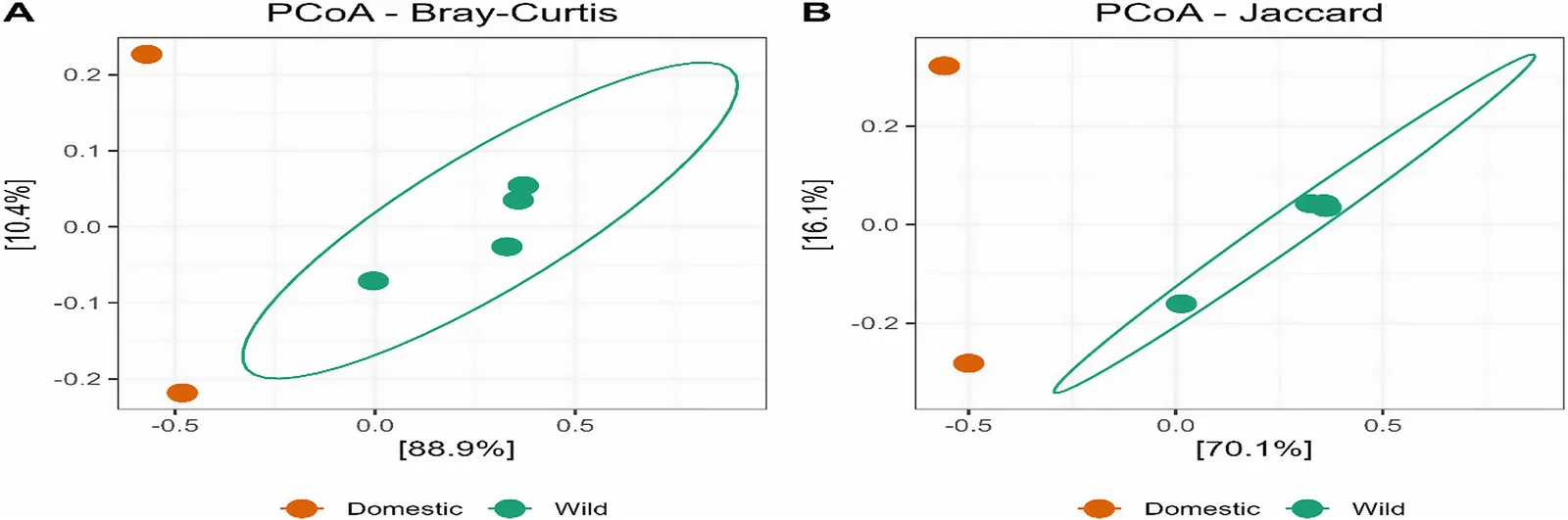
Principal coordinates analysis (PCoA) of microbial community composition in six pooled samples (E1-E6) based on (A) Bray-Curtis dissimilarity and (B) Jaccard dissimilarity. Percentages on the axes show the percentage of variation accounted for by each axis. Domesticated samples (E1 and E2) are represented by orange points and wild-type samples (E3-E6) are represented by green points.

A similar pattern was seen in the Jaccard ordination (Fig 3B). The first two axes accounted for 70.1% and 16.1% of the variation, respectively. E1 and E2 were located to the left of E3 to E6 on axis 1, but were separated on axis 2. In this ordination, the wild samples E4, E5 and E6 were relatively close to each other.

The taxonomic profiles at the phylum level revealed that the relative abundance of Cyanobacteria was about 90% in E1, 80% in E2 and 37% in E3 (Fig 4). Cyanobacteria were present in E4, E5 and E6 in small amounts. Pseudomonadota were abundant in E4, E5 and E6, and made up a significant percentage of E3. These profiles coincided with the separation of samples along axis 1 in the Bray-Curtis ordination.

**Fig 4:**
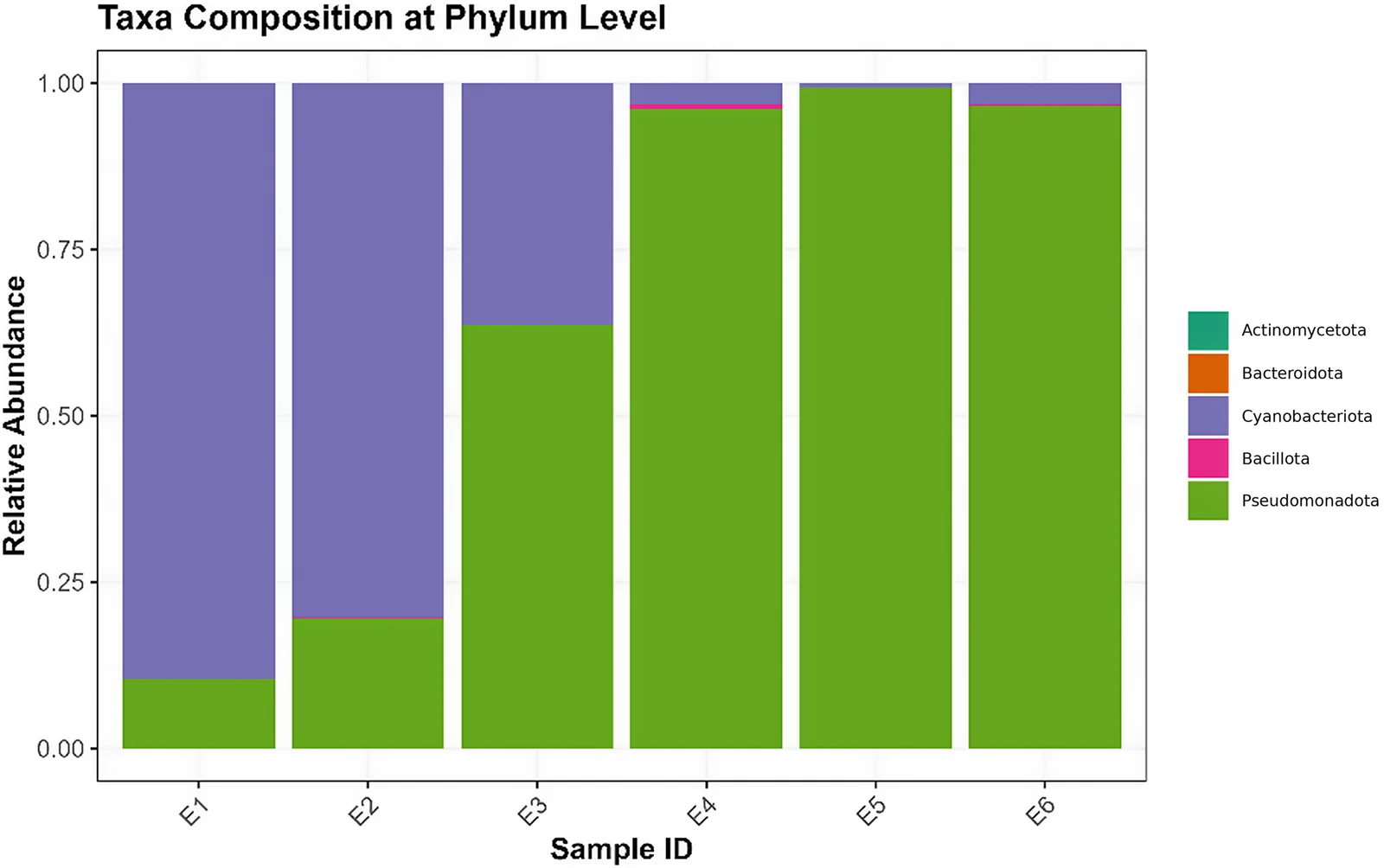
Relative abundances of bacterial phyla in domesticated and wild *Crotalaria* plants. E1 and E2 represent domesticated *Crotalaria* samples and E3, E4, E5 and E6 represent wild samples.

### 3.4. Bacterial taxonomic composition of *Crotalaria* endophytes

A consensus BLAST method was used to classify the bacterial sequences that were obtained from the samples by comparing them to the SILVA reference database. Interactive bar graphs illustrating the relative abundances of different taxa across various samples at multiple taxonomic levels were generated by the analysis. The sequences belonged to five phyla, of which Pseudomonadota was the most prevalent phylum detected (64.27%) followed by Cyanobacteriota (35.5%). Bacillota, Bacteroidota and Actinomycetota were present at minor concentrations (<5% each). Among the domesticated samples, Cyanobacteriota was the most dominant phyla at 84.9%, followed by Pseudomonadota (15.1%) and Bacillota (0.06%), while in wild samples Pseudomonadota was the dominant phyla at 88.9% followed by Cyanobacteriota (10.8%) and Bacillota (0.3%). The phylum Bacteroidota was reported only in domesticated sample E1, while the phylum Actinomycetota was only reported in wild sample E4 (Fig 4).

The sequences belonged to 20 families with the most abundant being *Enterobacteriaceae* at 81.06% followed by *Pseudomonadaceae* at 7.50% and *Xanthobacteraceae* at 1.44%. At the genus level, the sequences reads were taxonomically assigned to 30 genera, the majority of which were found in both wild and domesticated samples. Overall, the genus *Enterobacter* recorded the highest relative abundance of 72.4%, distantly followed by *Pseudomonas, Acinetobacter* and *Bradyrhizobium* with relative abundance of 14.8%, 3.5% and 2.7% respectively. Samples E3, E4, E5 and E6, all belonging to the wild group type of endophytes presented the highest relative abundances of *Enterobacter*, while domesticated sample E1 exhibited the lowest relative abundance of *Enterobacter* (12.5%). The genera *Phenylobacterium, Stenotrophomonas* and *Paenarthrobacter* were the least represented overall (Fig 5).

**Fig 5:**
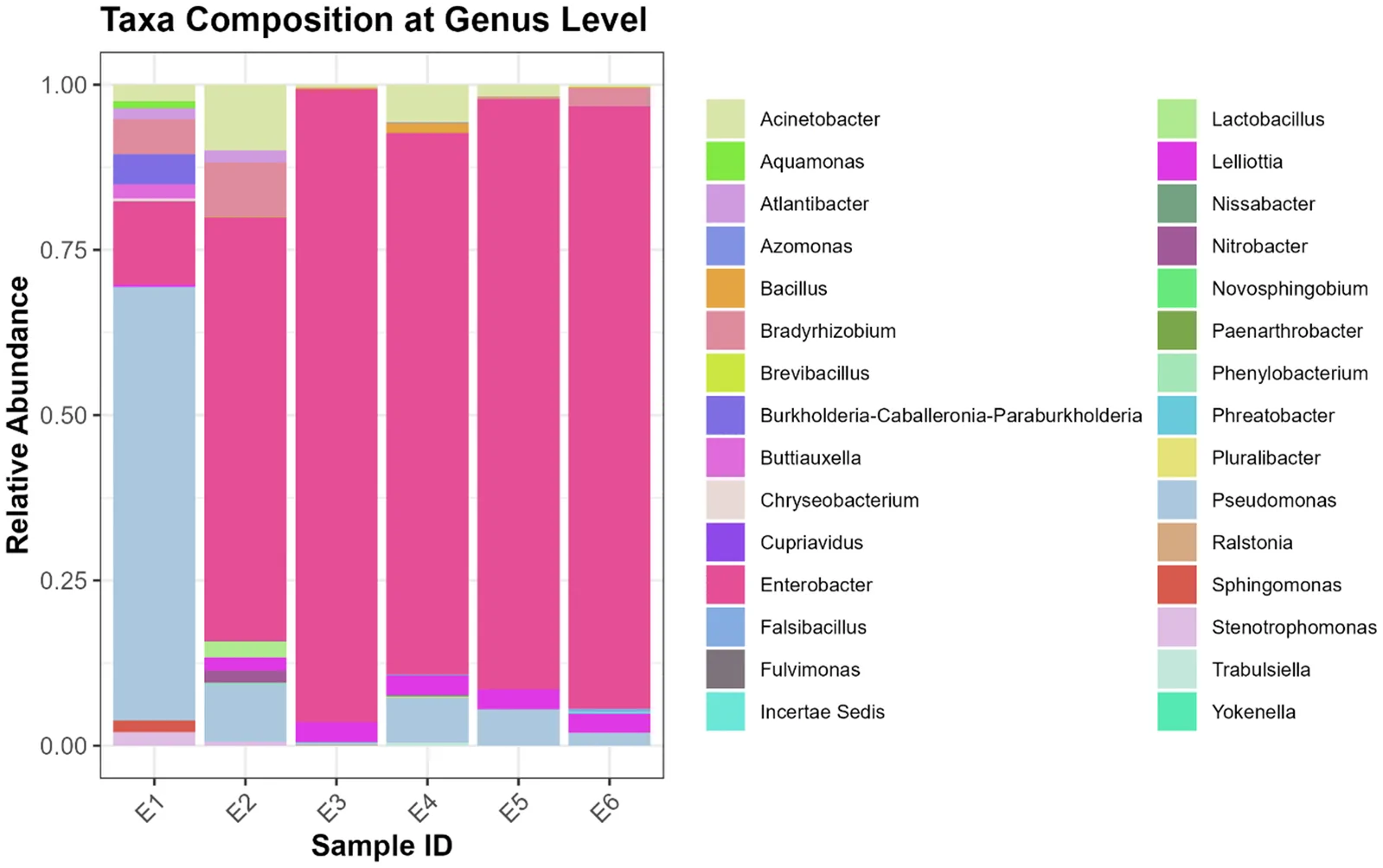
Relative abundances of different bacterial genera in both domesticated and wild *Crotalaria* plants. E1 and E2 represent domesticated *Crotalaria* samples and E3, E4, E5 and E6 represent wild samples.

Twenty (20) genera were reported in wild *Crotalaria* samples. The dominant genera (average relative abundance ≥ 0.5%) were *Enterobacter* (89.4%), *Pseudomonas* (3.7%), *Lelliottia* (2.96%), *Acinetobacter* (2.09%) and *Bradyrhizobium* (0.69%), which collectively accounted for 98.91% of the total endophytic bacterial community of the wild samples. Additional genera reported, but in lower relative abundances included: *Brevibacillus* (0.39)*, Bacillus* (0.17)*, Falsibacillus* (0.17)*, Trabulsiella* (0.10)*, Burkholderia-Caballeronia-Paraburkholderia* (0.01)*, Atlantibacter* (0.02)*, Ralstonia* (0.013)*, Phreatobacter* (0.01)*, Nissabacter* (0.07)*, Pluralibacter* (0.03)*, Yokenella* (0.01)*, Azomonas* (0.01)*, Paenarthrobacter* (0.004)*, Stenotrophomonas* (0.002) and *Phenylobacterium* (0.001)*.* Eighteen (18) bacterial genera were detected in the domesticated *Crotalaria* samples. The dominant genera (average relative abundance ≥ 0.5%) included *Enterobacter* (38.3%)*, Pseudomonas* (37.2%)*, Bradyrhizobium* (6.7%)*, Acinetobacter* (6.2%)*, Burkholderia-Caballeronia-Paraburkholderia* (2.3%)*, Atlantibacter* (1.8%)*, Stenotrophomonas* (1.4%)*, Lactobacillus* (1.2%)*, Lelliottia* (1.11%)*, Buttiauxella* (1.07%)*, Nitrobacter* (0.94%)*, Sphingomonas* (0.9%) and *Aquamonas* (0.5%)*.* The sum of the relative abundances of the 13 genera represented 99.54% of the endophytic bacterial community in domesticated samples. Other genera that were reported at lower abundances were *Chryseobacterium* (0.2)*, Cupriavidus* (0.04)*, Novosphingobium* (0.07)*, Fulvimonas* (0.06) and *Brevibacillus* (0.06)*.*

Some endophytes were identified only in domesticated or wild samples. At the genus level, *Bacillus, Falsibacillus, Trabulsiella, Ralstonia, Phreatobacter, Nissabacter, Pluralibacter, Yokenella, Azomonas, Paenarthrobacter and Phenylobacterium* were detected only in wild samples, while *Lactobacillus, Buttiauxella, Nitrobacter, Sphingomonas, Aquamonas, Chryseobacterium, Cupriavidus, Novosphingobium* and *Fulvimonas* were detected only in domesticated samples. Genera of endophytic bacteria that were shared between wild and domesticated samples included *Enterobacter, Pseudomonas, Bradyrhizobium, Acinetobacter, Burkholderia-Caballeronia-Paraburkholderia, Atlantibacter, Stenotrophomonas, Lelliottia* and *Brevibacillus*

## 4. Discussion

The current study provides a high-resolution characterization of endophytic bacterial communities associated with domesticated and wild *Crotalaria* species. The study further reveals that domestication primarily restructures community composition rather than overall diversity. The recovery of 611 ASVs across five phyla, supported by well-saturated rarefaction curves, indicates sufficient sequencing depth that included majority of the endophytic bacterial diversity from the sampled *Crotalaria* plant tissues.

A key pattern emerging from the data is the decoupling of richness and evenness. Alpha diversity patterns revealed a paradox frequently reported in plant microbiome studies. In the present study domesticated plants exhibited marginally higher observed richness, whereas wild plants maintained greater evenness. This suggests two distinct assembly regimes. In cultivated systems, increased nutrient availability and reduced environmental heterogeneity are likely to relax selective constraints, thus permitting the coexistence of closely related taxa, that is the strain-level diversification while inflating richness through microdiversification. In contrast, wild environments appear to impose stronger ecological filtering, resulting in fewer but more evenly distributed taxa, consistent with long-term host-microbe co-adaptation in stable environments [8]. Similar trends have been reported to link agricultural inputs and host selection pressure that expand niche availability without necessarily increasing functional diversity [6,7]. However, the small sample size (n = 6) severely limited the statistical power, which likely explains the non-significant alpha diversity results despite visible trends. This leads to the hypothesis that domestication increases taxonomic richness primarily through strain-level diversification rather than expansion of higher-level phylogenetic diversity.

The ordination patterns suggest that there are differences in the microbial community composition between the six pooled samples. The separation of E1 and E2 from most of the wild samples in both PCoA plots is consistent with the differences in their taxonomic profile. The abundance of Cyanobacteriota is particularly high in E1 and E2, whereas Pseudomonadota are more abundant in E4, E5 and E6. The Bray-Curtis separation observed may be a result of differences in the abundance of dominant phyla. However, the ordination alone could not provide information on the contribution of individual phyla.

The microbial community composition of E4, E5 and E6 is relatively similar, while the microbial community composition of E1 and E2 is different, as suggested by the distance between these two samples. The similar arrangement in the Jaccard ordination indicates that the differences observed could be due to variation in taxon occurrence as well as relative abundance. But, from the plots alone, it is not possible to determine the extent of the contribution of each factor.

The Bray-Curtis ordination explained 99.3% of the variation in the first two axes, while the Jaccard ordination explained 86.2% of the variation. These percentages reflect the range of variation shown by the coordinates displayed and are not meant to show statistical significance. The patterns observed are descriptive only as the analysis is based on only six pooled samples, and no replication within morphotype. In addition, the domesticated and wild groups are not identical in species, and it is hard to differentiate effects due to domestication from species-specific effects. These results therefore show that there were compositional differences between the samples, but could not account for the role of domestication in these differences.

At the phylum level, the overwhelming dominance of Pseudomonadota reinforces their role as a conserved functional backbone of plant endospheres, particularly in legumes where they mediate nitrogen cycling, phytohormone production and plant growth promotion [30]. Several studies have reported Pseudomonadota as the most prevalent bacterial phylum in endophytic bacterial communities of numerous plant species, including legumes like *Crotalaria* [11,40,41]. A study by Ahoty *et al.* [29] reported Pseudomonadota as the predominant bacterial phylum isolated from roots, stems and leaves of *Crotalaria retusa*. Similarly, Sánchez-López *et al.* [27] documented a higher relative abundance of Pseudomonadota in the seed-endophyte microbiome of *Crotalaria pumila* across three successive generations. However, the pronounced shift toward Cyanobacteriota in domesticated samples is mechanistically more revealing. Cyanobacteriotal enrichment may reflect altered environmental conditions in managed systems, suggesting that domestication may modify the resource landscapes in ways that favour alternative functional guilds. This observation supports the hypothesis that agricultural management may reshape endophytic bacterial communities by altering resource availability, thereby selecting for taxa with distinct metabolic strategies.

At the family level, the dominance of Enterobacteriaceae in the present study is consistent with previous findings of seed-associated endophytic bacteria from *Crotalaria* species in Kenya, in which Enterobacteriaceae was the most abundant family recovered [42].

The enrichment of the genus *Pseudomonas* in domesticated samples is consistent with its well-documented role in agricultural systems as a plant growth-promoting and biocontrol genus [30]. Aserse *et al.* [28] reported *Pseudomonas* among the dominant gram-negative bacterial genera recovered from root nodules of *Crotalaria* species and other legumes in Ethiopia. Similarly, *Pseudomonas* was one the dominant endophytic bacteria genera isolated from *C. retusa* growing in Côte d’Ivoire [29]. More importantly, the presence of habitat-specific genera, such as *Bacillus* and *Paenarthrobacter* in wild samples, versus *Sphingomonas* and *Nitrobacter* in domesticated samples demonstrates strong environmental filtering and niche specialization. On the other hand, wild-specific taxa such as *Bacillus*, *Ralstonia*, and *Paenarthrobacter* likely reflect adaptation to resource-limited and heterogeneous environments, where stress tolerance and metabolic flexibility are critical. Together, these patterns support a dual-assembly model, in which a conserved core microbiome is supplemented by habitat-specific taxa shaped by environmental context. This supports the growing consensus that plant microbiomes are shaped by the combined effects of host genotype, soil microbiota and management practices [43]. *Methylobacterium*, which has been found to be dominant in *C. pumila* seeds [27], as well as *Rhizobium* and *Agrobacterium*, which have been recovered from *C. incana* nodules [28], were not detected in this study. This could be attributed to the removal of nodules prior to DNA isolation, which may explain the low representation of rhizobial genera.

Crucially, the coexistence of shared and habitat-specific taxa demonstrates that domestication reshapes microbial communities not through wholesale loss of diversity, but through reorganization of relative abundances and functional potential. This aligns with emerging evidence that plant domestication modifies microbiomes primarily by shifting ecological dominance patterns rather than eliminating taxa altogether. From an applied perspective, the unique taxa identified in wild *Crotalaria* suggest that these systems are reservoirs of underexplored microbial functions that may be lost or suppressed under cultivation.

## 5. Limitations and future studies

The samples size was small and unbalanced, i.e., two domesticated and four wild *Crotalaria* samples. This made it statistically difficult to detect differences between domesticated and wild groups. Further, DNA was pooled by morphotype, hence replication within groups could not be accounted for, and therefore variance estimation could not be done. Although plants were sampled at maturity (approximately three months of age), detailed soil ecotypes and field management conditions were not documented. These factors might have contributed to the observed endophytic bacterial community differences, but couldn’t be distinguished from the domestication effects. Therefore, future studies should incorporate larger and more balanced samples from different populations or environments. Additionally, the 16S rRNA amplicon sequencing approach offers limited resolution when dealing with closely related taxa and does not directly indicate functional potential. Whole-genome sequencing is therefore best suited to provide greater functional and taxonomic resolution. Lastly, inclusion of environmental variables like soil characteristics and nutrient availability in future studies will help differentiate the impact of domestication from those of the surrounding environment.

## 6. Conclusion

Domestication in *Crotalaria* does not diminish endophytic diversity but fundamentally reorganizes it, shifting microbiome assembly from environmentally driven to management-mediated regimes. The observed decoupling of richness and evenness, coupled with strong compositional divergence, indicates that cultivated systems promote strain-level expansion and convergence, whereas wild systems retain more balanced and ecologically filtered communities. The persistence of a conserved core microbiome alongside habitat-specific taxa supports a dual-assembly framework in which host selection and environmental context jointly shape microbial structure. The enrichment of distinct functional groups such as Cyanobacteriota in cultivated plants and stress-adapted taxa in wild hosts suggests that domestication alters resource landscapes and selects for different metabolic strategies. This reorganization rather than loss of microbial diversity has important implications. It may enhance certain agronomic functions while simultaneously constraining the broader functional reservoir maintained in wild populations. Critically, wild *Crotalaria* emerges as a valuable source of underexplored microbial diversity with potential applications in sustainable agriculture. However, translating these insights into function requires moving beyond taxonomic inference toward integrative approaches combining metagenomics, cultivation, and experimental validation. Without this, the ecological patterns described here remain descriptive rather than predictive.
